# Genome-Wide Comparative Profiles of Triterpenoid Biosynthesis Genes in Ginseng and Pseudo Ginseng Medicinal Plants

**DOI:** 10.3390/life13112227

**Published:** 2023-11-19

**Authors:** Jing Lu

**Affiliations:** Division of General Education, Seokyeong University, Seoul 02173, Republic of Korea; lujing@skuniv.ac.kr

**Keywords:** Pseudo ginseng, cytochrome, glycosides, ginsenosides, *Panax*, saponins

## Abstract

Saponin-rich medicinal plants, particularly ginseng and Pseudo ginseng, are valuable in traditional medical practice due to the presence of different saponins. These plants benefit from natural saponins/triterpenoids drugs, such as Ginsenosides, Gypenosides, Platycodins, and Lancemasides. Ginsenosides are highly required for research and functional materials preparation in industrial practices, and some compounds, like Compound-K, have been taken to human trials for various therapeutic applications. To elucidate the genes/transcripts profiles responsible for secondary metabolites and ginsenoside biosynthesis in Ginseng and Pseudo ginseng plant genomes, a comparative analysis was conducted in this study. Nine plant genomes with a 99% BUSCO completeness score were used, resulting in 49 KEGG secondary metabolite pathways, 571 cytochromes genes with 42 families, and 3529 carbohydrate genes with 103 superfamilies. The comparative analysis revealed 24 genes/transcripts belonging to the CYP716 family, which is involved in the ginsenoside biosynthesis pathway. Additionally, it found that various ginsenosides demonstrated strong binding affinity with twelve targets, with ginsenoside Rg3, Rg2, Rh1, Rh5, F3, Rh9, Panaxadione, Protopanaxatriol, Floral ginsenoside C, and Floral ginsenoside E exhibiting the highest binding affinities with the tested enzymes. Since these groups of enzymes are not yet fully characterized for Pseudo ginseng plants in the interconversion of triterpenoids, this comparative bioinformatics analysis could aid experimentalists in selecting and conducting characterization with practical knowledge.

## 1. Introduction

Ginseng, a non-model plant, is recognized as an adaptogen within the Panax genus in the *Araliaceae* family, encompassing 15 species and 7 subspecies. The term “*Panax*” originates from the Greek word “panacea”, indicating a universal remedy. Notably, the anthropomorphic root of *Panax ginseng* stands out for its medicinal attributes and shares a homologous genome size correlation with the human genome. The usage of ginseng is very prevalent in traditional Chinese medicine (TCM)- and oriental medicine (OM)-based health supplement industries. The first generation of drug discovery was based on the alkaloid drugs that were isolated from medicinal plants. After that, the medicinal plant’s effectiveness was determined by its phytochemical ingredients [1]. However, converting traditional medicine formulations to modern medicine has always been a challenge. This is because identifying the active ingredients of traditional medicine has been a long-standing problem. Nevertheless, modern evidence-based “high-throughput” technologies, especially “genome-wide” omics technologies, have recently paved the way for exploring the hidden nature of medicinal plants [2]. The ginseng medicinal plant has been the subject of over 10,000 research articles and thousands of patents for its various formulations and therapeutic phytochemicals. These articles can be classified into four broad perspectives: (1) Identifying and enhancing the ethnopharmacological properties; (2) Improving the plant cultivation process and biomass production in a short time; (3) Phytochemical conversion/synthesis; and (4) Identifying adulteration in ginseng products [3]. Among these, ginsenoside’s phytochemical conversion and synthesis are significant parts of ginseng research. The research mainly focuses on the cytochrome and carbohydrate enzymes from the plant and microbes from food and soil sources.

Ginseng has been used for centuries in traditional Chinese medicine and oriental medicine due to its yin–yang properties. In 1854, a German scientist named Garrigues isolated the Panaquilon chemical component from *Panax* plants, leading to the discovery of around 330 ginsenosides [4,5,6,7]. These triterpenoid chemical components are mainly found in the *Panax* family and have a dammarane backbone moiety with an array of glycans in their functional groups [8]. Some of these components are naturally occurring and called significant ginsenosides, while others are converted forms, known as minor ginsenosides [9,10]. However, major ginsenosides are not absorbed into our bloodstream because the intestinal bacterial population converts them into minor ginsenosides. Therefore, the pharmaceutical industry uses various natural nonpathogenic microbes to convert ginsenosides, primarily for removing glycans present in the backbone moiety [11]. One such ginsenoside is Compound-K, which has undergone clinical studies up to the human trial stage [12]. Ginsenosides are similar to cardiac-glycosides drugs, such as Deslanoside and Acetyldigitoxin, which have been used in pharmaceuticals. Additionally, ginsenosides are used as supplements to treat various hormone imbalances in humans [13]. The conversion of ginsenosides in microbiomes is mainly observed by cytochrome enzymes, and various cytochromes in *Panax* family plants characterize the ginsenoside biosynthesis pathways [14,15].

It is not just ginseng plants that are widely valued in the market. Pseudo ginseng, which is more affordable due to its wider accessibility, has also gained popularity. One such example is *Gynostemma pantaphyllum*, which is often referred to as the “poor man’s ginseng”. This plant contains triterpenoids called gypenosides, which are similar to ginsenosides (Rg3, Rc, Rd, MRb1, MRd, F2, Rb3, and Rb1) and are widely used in green tea around the world [16,17]. Due to the high demand for ginsenosides in the pharmaceutical industry, bacterial enzymes convert gypenosides and other gypenosides to ginsenosides. This has led to an increase in attention towards *G. pantaphylum* in medicinal plant research [17]. 

Codonopsis [18] and platycodons [19] roots are also considered poor man’s ginseng due to their similar root morphologies to ginseng. These roots contain triterpenoids such as Platycodins and Lancemasides, which are similar to ginsenoside Ro, making them beneficial in traditional medicines [20,21]. To understand the diversity of cytochrome and carbohydrate enzymes present in these plants and their triterpenoid biosynthesis pathways, a comparative genome/transcriptome analysis was conducted. This analysis aimed to harness the benefits of these enzymes in an in vitro yeast model to enhance the production of various ginsenosides or triterpenoid glycosides for therapeutic applications, similar to opioids biosynthesis [22]. The ginsenoside biosynthesis pathway is believed to be downstream of the isoprenoid biosynthesis pathway rooted in squalene synthase. Various cytochromes and glycosyltransferase enzymes are then characterized for major ginsenoside isoform biosynthesis, while minor ginsenosides are artificially synthesized from microbial cytochrome enzymes. In this study, genome mining was used to profile cytochrome and glucosyl transferase and hydrolase enzymes from ginseng, Pseudo ginseng, and gut microbiota to aid experimentalists in choosing the enzymes for saponin/ginsenoside biosynthesis applications.

## 2. Materials and Methods

### 2.1. Genome and Completeness Assessment 

Nine assembled genomes (five species from Panax (i.e., *Panax ginseng* (PAGI0), *Panax notoginseng* (PANO0), *Panax japonicus* (PAJA0), *Panax stipuleanatus* (PAST0), and *Panax quinquefolius* (PAQU0) [14])) and Pseudo ginseng (*Codonopsis lanceolata* (COLA0) [23], *Platycodon grandiflorus* (PLGR0) [24], *Gynostemma pentaphyllum* (GYPE0) [25], and outlier *Daucus carota* (DACA0)) were selected and downloaded from the Refseq assembly database. The respective annotations were obtained from the corresponding authors via email request [14,23,24,25]. The assemblies conformed with the genome completeness assessment with BUSCO v5.0 with the embryophyta10.0 dataset [26]. The details are given in Supplementary Table S1.

### 2.2. Clustering of Proteome

We included the manually curated ginsenoside conversion microbial enzymes [27] and enzymes involved in the ginsenoside biosynthesis pathway from MetaCyc database [28], along with nine genome proteomes. Moreover, we obtained the KEGG secondary metabolite pathways from KEGG pathway database (https://www.genome.jp/kegg/pathway.html, accessed on 1 February 2023) and extracted respective protein sequences and other information, such as pathway name and KEGG orthologs ids, using Python scripts. Additionally, the coverage values for the pathway were calculated following the method described by Kim et al. [29], as follows:

$$\mathrm{Normalized}\;\mathrm{value}=\frac{\sum a}{b}\times 100$$

The normalized value was calculated as (number of KEGG orthologs (KO) that have similar transcripts to the reference transcriptome/total number of KO in each pathway) × 100. Furthermore, we downloaded the terpenoid biosynthesis pathway proteome from UniProt database for additional confirmations.

### 2.3. Cytochrome and Glycosyl Transferase/Hydrolyse Family Analysis

The complete transcripts were compared with the CYPED (https://cyped.biocatnet.de/, accessed on 1 February 2023) [30] database using the CD-HIT method to obtain the CYP family. The parameters C:70 and S:70 were used for this purpose. The selected family enzymes were aligned using MAFFT v7.2 with default parameters [31]. The multiple alignments with MAFFT with –auto parameter and the aligned file in PHYLIP format subjected to Gblock (-t = p-e = -gb1-b4 = 5-d = y) to reduce the noise in the multiple alignments to secure the highly conserved regions of the given protein sequences. Finally, the concatenated conserved blocks were subject to IQTree with option -m MFP (model finder plus) to generate a phylogenetic tree. The multiple alignments were initially corrected with [32] and were used for constructing a phylogenetic tree by IQ-TREE v2.0 [33]. Finally, the tree was imported to FigTree v1.4.3 (http://tree.bio.ed.ac.uk/software/figtree/, accessed on 1 February 2023) to obtain an image, which is shown in Figures 3–5 and S1.

### 2.4. Docking Assessment 

As per the genome assessment carried out in this study, seven enzymes from the CYP716 family, namely, CYP716A52, CYP716A12, CYP716AL1, CYP716A15, CYP716A17, CYP716A47, CYP716A53, were found to be involved in the secondary metabolites and ginsenoside biosynthesis pathways, with reference to the MetaCyc pathway database. Additionally, the study also profiled five UGT1 family carbohydrate enzymes, UGT71A27, UGTPg101, UGTPg100, UGT74AE2, and UGT94Q2, and more details about them are provided in Table 1. To assess the binding potential of these twelve predominant enzymes, a molecular docking analysis was conducted for saponin/ginsenoside biosynthesis applications. Around sixty-six selected ginsenosides were virtually screened against these selected enzymes. For the ginsenosides whose 3D structure was not available in PubChem, their structures were manually sketched in ACD ChemSketch [34] and converted to 3D structures for further analysis. All the compounds and reference compounds were converted to PDB format using Open Babel [35]. The ligand molecules were then processed and converted to the required pdbqt format using Autodock tools [36].

The 3D structures of all the selected proteins were available in their native forms and were downloaded from the RCSB PDB database [37] in the PDB format. The structure preparation process involved several steps, including deleting all water molecules and inhibitors (ligands), checking and repairing the missing atoms, and adding hydrogens and required charges using Autodock tools. The final file was saved in the required format (pdbqt) for docking analysis. All docking experiments were performed using AutoDock Vina [38]. The selected twelve targets were used for molecular docking of ginsenosides. The compounds were ranked based on their docking scores, which represent their binding energies. The ligand interactions with the active sites of the receptors were visualized using the academic version of PyMOL [39] (DeLano, 2) and BIOVIA Discovery Studio Visualizer (BIOVIA, Dassault Systèmes, https://discover.3ds.com/discovery-studio-visualizer-download, accessed on 1 February 2023). Two-dimensional figures were drafted using the same software, and 2D diagrams were generated to depict hydrogen bonds and hydrophobically interacting residues. Each ligand cluster was inspected for amino acids interacting with the ligand, hydrogen bonds (H bonds), and the specific atoms involved.

## 3. Results and Discussion

### 3.1. Comparative Genomes

**Figure 1:**
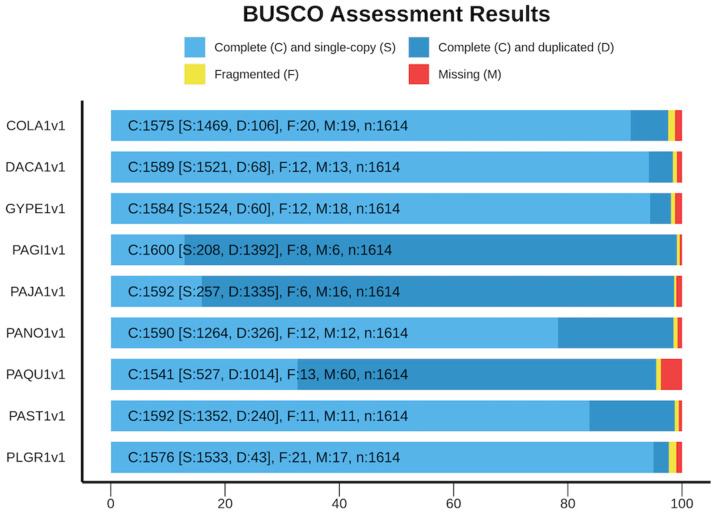
Summary of ginseng and Pseudo ginseng genome completeness BUSCO assessment.

The comparative analysis in this study included a total of five Panax genomes, three Pseudo ginseng plants (i.e., *C. lanceolata, P. grandiflorus*, and *G.pentaphyllum*), and an outgroup *D. carota* (Figure 1). The genome size of the selected plants varied, with *P. ginseng* having the largest genome size and *D. carota* having the lowest (Supplementary Table S1). The genome assembly assessment showed that almost 99% of genomes were assembled completely (Figure 1), ensuring that the genes/transcripts in this profile were completely covered and assessed for comparative profiles. Among the nine species, *P. quinquefolius* had the largest genome, while *D. carota* had the shortest. When looking at the BUSCO completeness assessments, *P. ginseng, P. japonicus, and P. quinquefolius* had more duplicated core genes than the others. This is due to the ploidy nature of the genus and the assembled genomes [23,24,40,41]. This may influence the high transcript isoforms in the gene/transcript numbers in genome annotation compared to others. It is worth noting that these Pseudo ginseng plants are widely used as an alternative for ginseng due to the saponin content present in their roots, making them popular in the traditional medicinal market [23,24,40]. 

### 3.2. Secondary Metabolite Biosynthesis 

**Figure 2:**
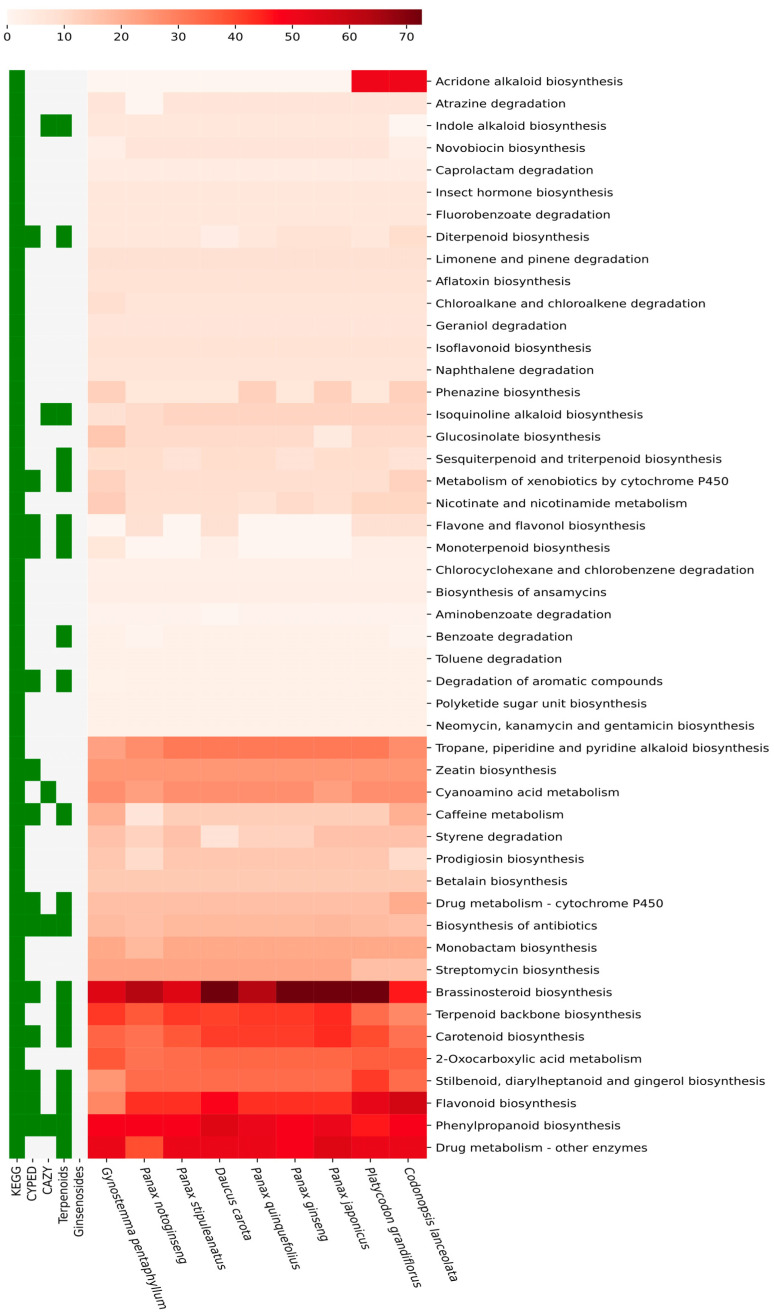
Complete secondary metabolite biosynthesis pathway coverage profiles with the reference of KEGG metabolic pathway database.

Medicinal plants are consumed for their medicinal properties and effective use in the therapeutic functional supplement food industries. However, traditional characterization methods that involve characterizing individual secondary metabolite components are limited in summarizing the whole array of secondary metabolites available in medicinal plants. In the genomic era, with the availability of individual plant genomes, it helps predict the available secondary metabolites through bioinformatics analysis (as shown in the graphical abstract). This study conducted a comparative genome analysis among Panax families and Pseudo ginseng plants using genome data from public repositories and published genome articles. Secondary metabolites were clustered with KEGG secondary metabolite pathways, resulting in 6933 sequences from nine genomes. Among them, 1178 and 1074 genes were present in *P. japonicus and P. ginseng*, respectively. The lowest 445 transcripts were present in *C. lanceolata*. The sequences were mapped to 49 secondary metabolite biosynthesis KEGG pathways (as shown in Figure 2). Using bioinformatics principles, this study provides a comprehensive analysis of the secondary metabolite biosynthesis pathways in these plants. In this study, it was observed that among the secondary metabolite biosynthesis pathways, the Acridone alkaloid biosynthesis pathway is rich in Pseudoginsengs, such as *C. lanceolata* and *P. grandiflorus,* compared to other ginsengs in the Panax family. The brassinosteroid biosynthesis pathway showed a difference in gene presence, with more than 70% of pathway genes present in carrot, *P. ginseng, P. japonicus, and P. grandiflorus* when compared to others. Similarly, the phenylpropanoid, terpenoid backbone, carotenoid, and flavonoid biosynthesis pathways covered more than 50% of KEGG pathways (as shown in Figure 2). This in silico pathway assessment could be a cost-effective approach for those plants with decoded genomes and aid the experimentalist in performing experiments for specific targets [42,43,44,45]. This approach could be a substitute for total secondary metabolite profiles experiments such as total phenolic content, total flavonoid content, and other subclasses of secondary metabolite quantifications.

### 3.3. Cytochrome Profiles

**Figure 3:**
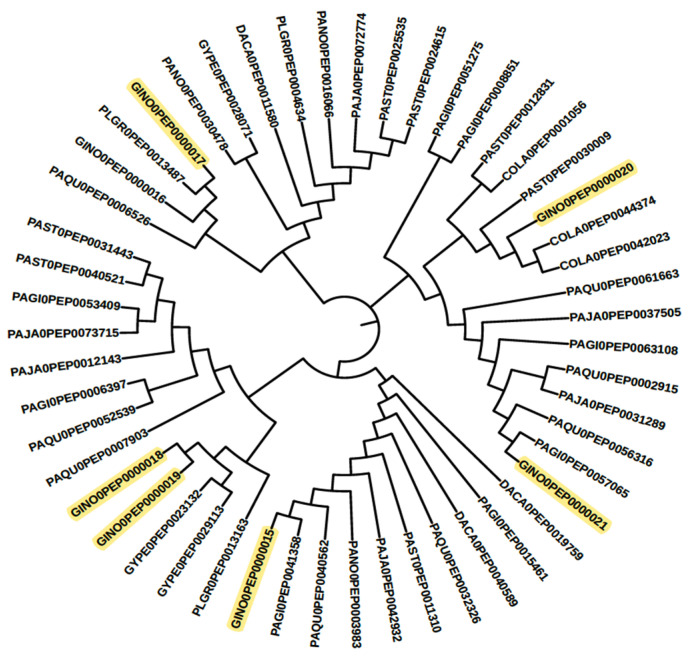
The phylogenetic tree for the Cytochrome Superfamily 716 proteins was constructed with reference to the MetaCyc ginsenoside biosynthesis pathway. In this tree, the key GINO0 represents the MetaCyc ginsenoside biosynthesis pathway enzyme sequence.

The advantage of “genome-wide” comparison is that it provides an overview of the selected targets/biosynthesis pathways from a desired plant. Earlier, a similar profiling process was conducted for cytochrome multifamily genes with expressed sequence tags (EST) in *P. ginseng* [46], which was later improved with next-generation sequencing through de novo transcriptome assemblies [47], and, finally, performed with chromosome-scale assembled genomes [14]. Limited CYPs were observed when EST and de novo transcriptome assemblies were used, as compared to whole-genome assemblies [14]. Only three clans of partial cytochrome, i.e., CYP71, CYP90, and CYP72, were identified when compared to whole-genome-based CYP profiles, which is a partial result [14,46,47]. This study is the first comparative CYP profiling study for Pseudo ginseng along with the Panax families. Through systematic bioinformatics analysis, as illustrated in Figure 1, forty-two cytochrome families were identified from *Panax* and other Pseudo ginseng plants. Among those, 16 cytochrome families were found to be involved in terpenoid biosynthesis, and 19 others were found to be involved in other secondary metabolite biosynthesis pathways. According to the MetaCyc pathway database, the CYP716 family enzymes are primarily characterized for ginsenoside biosynthesis. In total, twenty-four sequences from all selected genomes were plotted as a phylogenetic tree in Figure 3. As previously mentioned in Zang et al. [48], *Panax* plants were summarized into three groups based on their available ginsenoside profiles. However, due to the lack of enough datasets, a similar process has not yet been carried out for Pseudo ginseng plants. Therefore, in this study, *Panax* plants were considered as a model/reference for Pseudo ginseng to gain knowledge on triterpenoid biosynthesis pathway possibilities. These findings could be used to develop a similar yeast model for the industrial production of opioids cascade biosynthesis in one step [22]. For example, due to the long-life cycle of ginseng, raw material shortage is common in various industries. As a result, researchers are exploring the conversion of saponins from Pseudo-ginseng, such as ginsenosides from gypenosides, under laboratory conditions [49,50]. Our bioinformatics analysis identified CYP716A (CYP716A47 [50], CYP716A53v2 (protopanaxadiol to protopanaxatriol) [51], CYP716A52v2 (oleanane-type ginsenoside biosynthesis) [52], and CYP716A52v2 (oleanane-type ginsenoside biosynthesis) [53]) group enzymes from Pseudo ginseng, which are similar to the well-characterized CYP716A enzymes involved in the ginsenoside biosynthesis pathway (as shown in Figure 3). For instance, the sequence GINO0PEP0000017 is representative of the CYP716A47 family enzyme involved in the catalytic process of dammarenediol-II to protopanaxadiol in the ginsenoside biosynthesis pathway [51], and similar sequences are present in PLGR0 and PAQU0. Similarly, other CYP716 families in ginseng plants are not characterized in detail. Other sequences with high similarity may have similar characteristics, which need to be checked through experiments [54].

### 3.4. Carbohydrate Enzymes Profiles

The biosynthesis of glycosides such as Ginsenosides, Gypenosides, Platycodins, and Lancemasides in plants is diverse, and subsequent glycosylation enzymes contribute to the synthesis of various glucoside secondary metabolites. Various carbohydrates such as UDP-alpha-D-glucose, UDP-GluA, UDP-Xyl, UDP-Gal, UDP-Arap, UDP-Araf, and UDP-Rha are present in these terpenoid backbone moieties due to the presence of various carbohydrate enzymes. Since around 300 ginsenoside [27] and 200 gypenoside isoforms [17] have been identified, most isoforms vary based on the different types and numbers of carbohydrates in the backbone moiety. In ginsenosides, most of the UDP-sugars are attached to the functional group in C-6 and C-20 in PPT-Type, C-3 and C-20 hydroxyl groups in PPD-type, and C-3 hydroxyl and C-28 carboxyl groups in OA-type ginsenosides. Profiling the carbohydrate enzymes, as explained in the Materials and Methods, resulted in 3529 sequences belonging to carbohydrate enzymes, which belong to 103 superfamilies of carbohydrate enzymes in the CAZY database.

**Figure 4:**
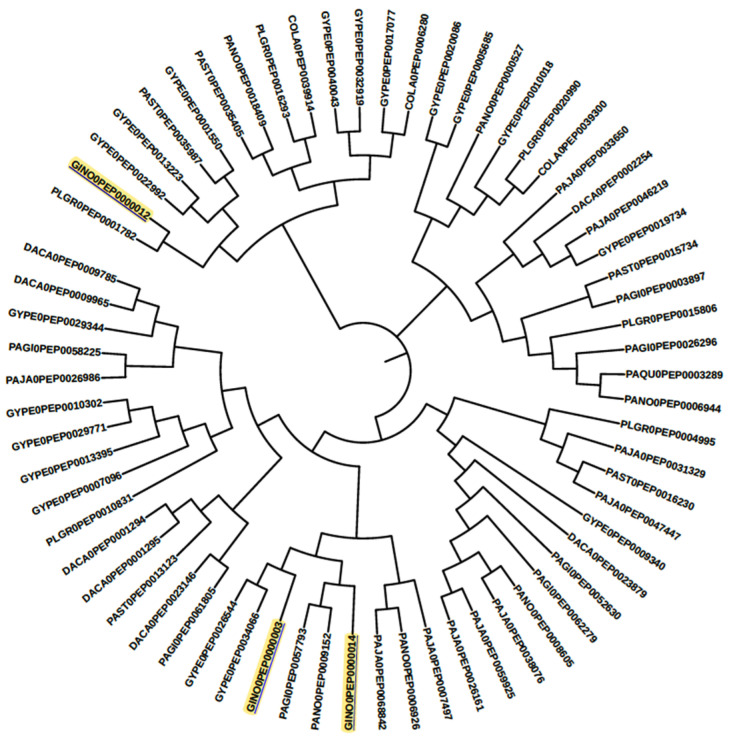
The phylogenetic tree for the carbohydrate enzyme superfamily GH1 proteins is presented here, along with the reference of the MetaCyc ginsenoside biosynthesis pathway. The GINO0 key represents the enzyme sequence for the MetaCyc ginsenoside biosynthesis pathway.

**Figure 5:**
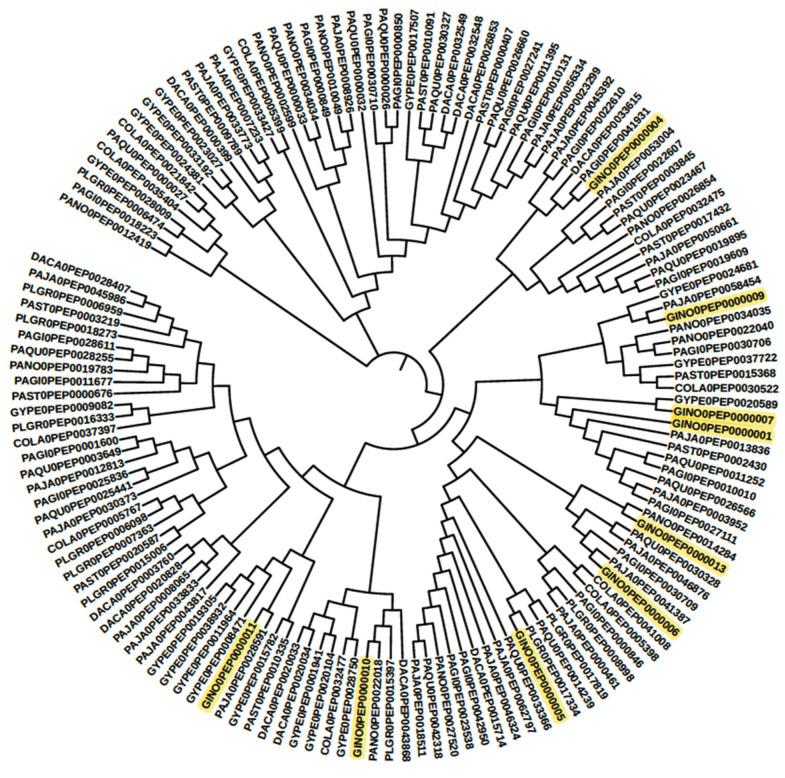
The phylogenetic tree for the carbohydrate enzyme superfamily GH3 proteins is presented here, along with the reference of the MetaCyc ginsenoside biosynthesis pathway. The GINO0 key represents the enzyme sequence for the MetaCyc ginsenoside biosynthesis pathway.

It is worth noting that there are several subcategories of enzymes that play important roles in plant physiology. For example, the Auxiliary activity family enzymes (AA0, 1, 5, 6) are widely present in all genomes and are involved in handling antioxidants. Carbohydrate-binding modules (CBM13, 43, 45, 48, 50, and 57) are also present in all genomes, as are members of the carbohydrate esterase family (CE11, CE8, 13). Glycoside Hydrolase (GH1 and GH3) families are involved in ginsenoside biosynthesis. The glycosyl-transferase family (which includes 41 families) is also important, with GT1 being characterized for ginsenoside biosynthesis. Three superfamilies within this group are involved in terpenoid biosynthesis (GT1, GT2, GT4). It is interesting to note that there are two families of pectin lyase present (PL1, PL4), and among the *Panax* family plants, UGT71-100 is the most prevalent. Within this family, the UGT1 group of enzymes is the largest. Enzymes from the AA0, GH3, and GH1 families are involved in secondary metabolic pathways, while GT1,2,4 and GH1 are involved in terpenoid biosynthesis. Specifically, in the ginsenoside biosynthesis pathway, the GH1, GH3, and GT1 family enzymes are involved, as per the MetaCyc pathway database. Interestingly, the enzyme beta-glucosidase is also involved in the process of converting saponins from Pseudo ginseng to ginsenoside, similar to cytochromes [27]. However, many of these sequences have not been characterized in detail for triterpenoid biosynthesis. The phylogenetic tree in Figure 4, Figure 5 and Figure S1 plots these sequences.

### 3.5. Ginsenoside Interactions with Genes in Ginsenosides Biosynthesis Pathway

**Figure 6:**
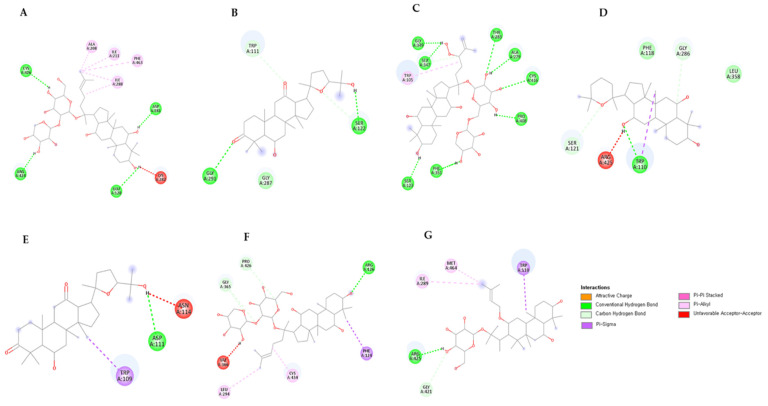
2D interaction diagram of seven CYP716 family enzymes with the highest ranking ginsenosides. (**A**) CYP716A12, (**B**) CYP716A52, (**C**) CYP716A53, (**D**) CYP716A15, (**E**) CYP716AL1, (**F**) CYP716A47, and (**G**) CYP716A17.

**Figure 7:**
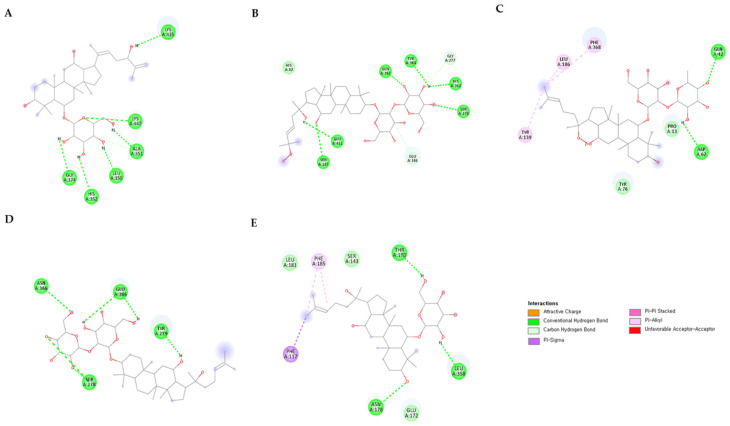
2D interaction diagram of five UGT1 family enzymes with the highest ranking ginsenosides. (**A**) UGTPg100, (**B**) UGTPg101, (**C**) UGT74AE2, (**D**) UGT71A27, and (**E**) UGT94Q2.

Based on molecular docking studies, it was found that most of the ginsenosides tested in this study showed highly competitive binding affinity with all twelve targets. The binding energies ranged from −11.5 kcal/mol^−1^ to −5.9 kcal/mol^−1^, which is significant. Further analysis of the ligand with the highest binding affinity for the twelve targets was conducted to assess the molecular interaction. You can find more details about the binding energies of the targets and ligands in the Supplementary Table S2. According to the results, ginsenoside Rg3, Rg2, Rh1, Rh5, F3, Rh9, Panaxadione, Protopanaxatriol, Floral ginsenoside C, and Floral ginsenoside E showed the highest binding affinity with the enzymes tested. Protopanaxatriol had the highest binding energy with the enzyme CYP716A15, which was −11.5 kcal/mol^−1^. It interacted with the TRP110 amino acid residue of the target. Ginsenoside Rg3, on the other hand, had a higher affinity of −11.1 kcal/mol^−1^ with the UGT71A27 enzyme, and interacted with the ASN366, GLU386, TYR279, and SER278 amino acid residues. The 2D structures, binding scores, and interactions of each enzyme’s top compounds are provided in Table 2 and Table 3 and Figure 6 and Figure 7.

## 4. Conclusions

The biosynthesis of ginsenosides comes from two significant steps, i.e., formation and modification of ginsenoside backbone moiety and carbohydrates ligation and hydrolysis processes by cytochrome and carbohydrate enzymes. In the ginseng plants, the major ginsenosides are highly dominating, and the minor ginsenosides are absent or low in quantity. As explained earlier, to enhance the production of minor ginsenosides, the enzymes are taken from the microbes of the food products and ginseng plant rhizosphere. When it comes to carbohydrates, the difference in sidechains majorly takes place by the difference in cytochrome and carbohydrate enzymes present in plants. As observed in the cytochromes profile, CYP716, already well characterized for ginsenoside biosynthesis, is present in all nine plants in our study. Furthermore, two superfamilies of UDP-dependent glycosyltransferases (UGTs) are widely present in all nine plants, similar to enzymes in the ginsenoside biosynthesis pathway. The superfamily GT1 contains the plant subfamily 71–100, which contributes to inverting the catalytic function of carbohydrates. The molecular docking studies also reveal that the ginsenosides interact with the target enzymes with higher binding affinity, which require further in-depth experimental validation such as experimental assays like enzyme activity tests and targeted metabolomics, which can confirm predicted functions. CRISPR/Cas9 gene editing can be employed to validate specific gene roles. Further, integrating these experiments with computational predictions enhances understanding and aids in developing new production strategies for bioactive compounds.

## Figures and Tables

**Table 1:** Enzymes involved predominantly in secondary metabolite biosynthesis.

S.No	Uniprot ID	Gene	Protein_Name
1	I7C6E8	CYP716A52v2	Beta-amyrin 28-monooxygenase
2	Q2MJ20	CYP716A12	Beta-amyrin 28-monooxygenase
3	I1TEM3	CYP716AL1	Cytochrome P450
4	F1T282	CYP716A15	Beta-amyrin 28-monooxygenase
5	F1T283	CYP716A17	Beta-amyrin 28-monooxygenase
6	H2DH16	CYP716A47	Dammarenediol 12-hydroxylase
7	I7CT85	CYP716A53v2	Protopanaxadiol 6-hydroxylase
8	A0A0A7HB61	UGT71A27	UDP-glycosyltransferase 71A27
9	A0A0K0PVM5	UGTPg101	UDP-glycosyltransferase 101
10	A0A0K0PVW1	UGTPg100	UDP-glycosyltransferase 100
11	A0A0A6ZFR4	UGT74AE2	UDP-glucosyltransferase 74AE2
12	A0A0A6ZFY4	UGT94Q2	UDP-glucosyltransferase 29

**Table 2:** Interaction of compounds with amino acid residues of CYP716 family enzymes.

Target Enzyme	Compound	Binding Energy (kcal/mol)	H-Bond Interactions	Other Interactions	No. of H-Bond
CYP716A12	Ginsenoside F3	−10.3	CYS426; ARG424; GLU128; ASP281	LYS282; ALA208; ILE211; PHE463; ILE288	4
CYP716A52	Panaxadione	−10.9	GLY291; SER122	TRP111	2
CYP716A53	Floral ginsenoside C	−10.7	GLY349; SER347; SER123; PHE351; THR283; ALA279; CYS416; PRO408	TRP105	8
CYP716A15	Protopanaxatriol	−11.5	TRP110	SER121; GLY286; ARG425	1
CYP716AL1	Panaxadione	−10.7	ASP111	ASN114; TRP109	1
CYP716A47	Ginsenoside F3	−9.6	ARG126	GLY365; PRO426; VAL366; LEU294; CYS434; PHE124	1
CYP716A17	Ginsenoside Rh9	−10.7	ARG425	GLY421; ILE289; MET464; TRP110	1

**Table 3:** Interaction of compounds with amino acid residues of UGT1 family enzymes.

Target Enzyme	Compound	Binding Energy (kcal/mol)	H-Bond Interactions	Other Interactions	No. of H-Bond
UGTPg100	Ginsenoside Rg5	−10.4	LYS435; LYS447; ALA351; GLY374; LEU350; HIS352	-	
UGTPg101	Floral ginsenoside E	−10.7	GLU411; SER187; GLN387; TYR384; HIS362; SER278	GLY277; GLU386	6
UGT74AE2	Ginsenoside Rg2	−9.2	GLN42; ASP62	TYR139; LEU186; PHE368	2
UGT71A27	Ginsenoside Rg3	−11.1	ASN366; GLU386; TYR279; SER278		4
UGT94Q2	Ginsenoside Rg	−10	THR170; LEU358; ASN178	PHE185; PHE117	3

## Data Availability

The proposed sequence files are deposited in the figshare repository under the https://doi.org/10.6084/m9.figshare.24590688.v1. The genome and proteome sequences used in this study were obtained from the respective authors, as outlined in the method section. You can find the GenBank identifier for those sequences in Supplementary Table S1.

## Supplementary Materials

The following supporting information can be downloaded at: https://www.mdpi.com/article/10.3390/life13112227/s1, Figure S1: The phylogenetic tree for the carbohydrate enzyme superfamily GT1 proteins along with the reference of metacyc ginsenoside biosynthesis pathway; Table S1; Table S2.
