# Supplementary material for: Genome-Wide Comparative Profiles of Triterpenoid Biosynthesis Genes in Ginseng and Pseudo Ginseng Medicinal Plants
Source: Life (Basel). 2023 Nov 19;13(11):2227. doi: 10.3390/life13112227 (PMC10672587; doi:10.3390/life13112227)
Supplement: Supplementary file 1 [file life-13-02227-s001.zip › Supplementary_figures.pdf]

Genome wide comparative profiles of triterpenoid biosynthesis genes in ginseng and pseudo ginseng medicinal plants

Jing Lu

Division of General Education, Seokyeong University, Republic of Korea.

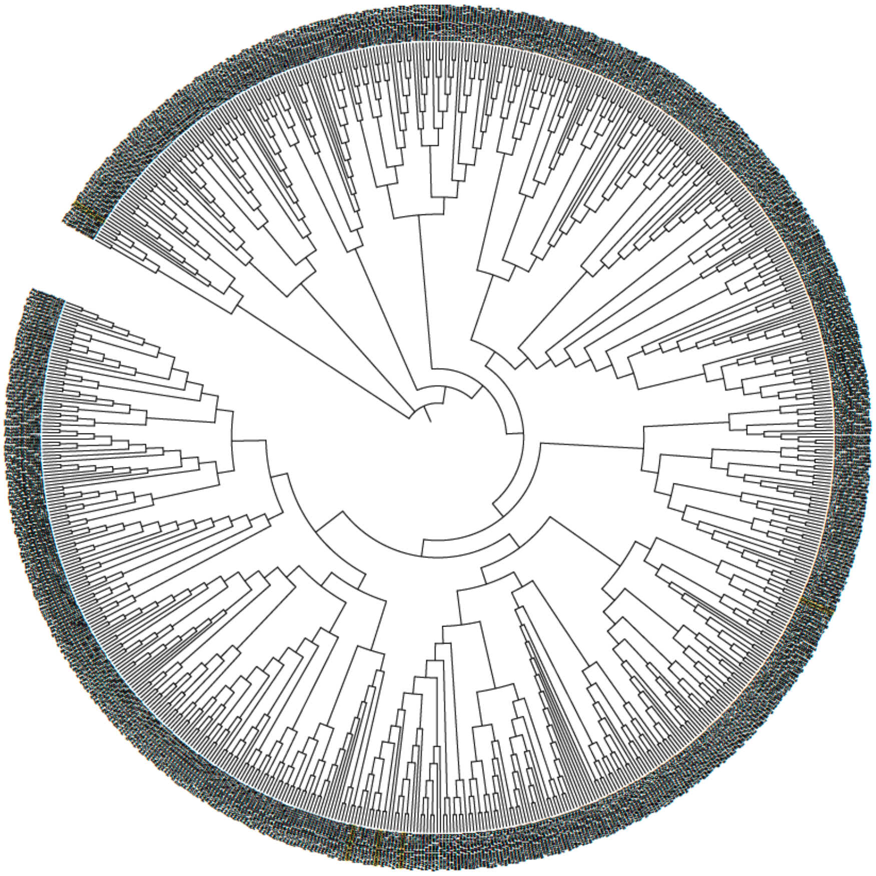

Figure S1: The phylogenetic tree for the carbohydrate enzyme superfamily GT1 proteins along with the reference of metacyc ginsenoside biosynthesis pathway. Here GINO0 key represent the metacyc ginsenoside biosynthesis pathway enzyme sequence.
